# Fifteen Years of Health-and-Environment Research in Slovenia

**DOI:** 10.2478/sjph-2026-0001

**Published:** 2026-03-01

**Authors:** Tanja Carli, Andreja Kukec, Lijana Zaletel-Kragelj, Ivan Eržen

**Affiliations:** National Institute of Public Health, Trubarjeva cesta 2, 1000 Ljubljana, Slovenia; University of Ljubljana, Faculty of Medicine, Department of Public Health, Zaloška cesta 4, 1000 Ljubljana, Slovenia

**Keywords:** Environmental medicine, Epidemiology, Methods, Research, okoljska medicina, epidemiologija, metode, raziskovanje

## Abstract

The environment in which we are born, live, work, and age is an important determinant of human health. This review summarises key epidemiological studies conducted over the past 15 years at the Department of Public Health, Faculty of Medicine, University of Ljubljana, in collaboration with experts in public health, clinical medicine, and other health and environmental disciplines. Methodological approaches for linking health and environmental data, including advanced spatial epidemiological methods to assess the impact of ambient air pollutants on respiratory diseases and diabetes, were highlighted. Considering the observed health outcomes among children and adolescents, new tools were developed and validated to estimate the prevalence of asthma and sleep-related problems among youths. In adults, the review examines the lifetime prevalence of first and recurrent systemic allergic reactions to bee venom among beekeepers, emphasising the need for effective risk-management strategies. Overall, the studies demonstrated the value of integrating health and environmental data to better understand and address public health challenges in Slovenia. The findings underscore the importance of interdisciplinary collaboration in developing evidence-based public health policies to mitigate environmental health risks and promote a high quality of life for present and future generations.

## Izvleček

Okolje, v katerem se rodimo, živimo, delamo in staramo, je pomembna determinanta zdravja. Namen prispevka je predstaviti ključne epidemiološke raziskave s področja zdravja in okolja v zadnjih 15 letih, zasnovane in izvedene na Katedri za javno zdravje, Medicinske fakultete Univerze v Ljubljani, v sodelovanju s strokovnjaki s področja javnega zdravja, klinične medicine ter drugih zdravstvenih in okoljskih ved. Izpostavljeni so metodološki pristopi za povezovanje zdravstvenih in okoljskih podatkov z uporabo naprednih prostorskih epidemioloških metod za ocenjevanje vpliva onesnažil v zunanjem zraku na bolezni dihal in sladkorno bolezen. V populacijski skupini otrok in mladostnikov pomemben metodološki doprinos predstavlja razvoj in validacija novih orodij za oceno prevalence astme in motenj spanja. V odrasli populaciji pregled izpostavlja nacionalno raziskavo med slovenskimi čebelarji, kjer je bila z razvojem in validacijo novega orodja ocenjena prevalenca prve in ponovne sistemske alergijske reakcije po piku čebele. Raziskave dokazujejo pomembnost povezovanja zdravstvenih in okoljskih podatkov z namenom boljšega razumevanja in reševanja izzivov na področju javnega zdravja. Ugotovitve poudarjajo pomembnost interdisciplinarnega sodelovanja pri oblikovanju na dokazih temelječih javnozdravstvenih politik za zmanjšanje okoljskih tveganj in spodbujanja visoke kakovosti življenja sedanjih in prihodnjih generacij.

## 1 INTRODUCTION

The environment in which we are born, live, work, and age is an important determinant of human health. A clean and healthy environment supports well-being, whereas environmental contamination poses substantial risks (1). Air pollution and extreme weather events driven by climate change are among the most significant environmental threats affecting human health. However, environmental hazards do not affect all communities equally, as socially disadvantaged and otherwise vulnerable groups disproportionately bear the burden of these risks. To address these disparities, global policy frameworks, including the 2030 Agenda for Sustainable Development and the Ostrava Declaration, provide strategic guidance for action in the field of health and environment. The 2030 Agenda, adopted in 2015 by all United Nations Member States, outlines 17 Sustainable Development Goals that link a healthy natural environment with improved wellbeing across all age groups, with particular emphasis on reducing maternal and child mortality. At the broader European level, these priorities are reflected in the Ostrava Declaration, adopted in 2017 within the World Health Organisation (WHO) framework (2). Putting these commitments into national action, Slovenia adopted the Resolution on the National Environmental Protection Programme 2020–2030 (3), aiming to ensure that preserved nature and a healthy environment support a high quality of life for present and future generations.

Designing evidence-based health and environmental policies requires collaboration across a wide range of disciplines and sectors, including the education system. This review of research aims to present key environmental studies designed and conducted at the Department of Public Health, Faculty of Medicine, University of Ljubljana, in collaboration with experts in public health, clinical medicine, and other health and environmental disciplines, published between 2010 and 2025.

## 2 METHODOLOGICAL APPROACHES FOR LINKING HEALTH AND ENVIRONMENTAL DATA

Methodologies for assessing spatial relationships between health outcomes and environmental risk factors have been recommended by the WHO for two decades (4, 5). This population-level approach—used to examine the association between respiratory diseases in children and adolescents, and particulate matter (PM_10_)—was applied in the Zasavje region using two complementary study designs: an ecological time-series study (6) and a spatial epidemiological study (7). The developed methodology was later used across Slovenia within the European project MED-HISS (Engl. *Mediterranean Health Interview Surveys Studies*), coordinated by the National Institute of Public Health of Slovenia. As part of this project, an ecological analysis was conducted to assess the association between selected health outcomes and ambient air quality at the municipal level (8). The methodological approach was subsequently used in studies investigating associations between observed health outcomes (daily counts of deaths from respiratory diseases (9) and primary health care consultations for diabetes (10)) and exposure to ultrafine particles in ambient air in Ljubljana.

## 3 OBSERVED HEALTH OUTCOMES

### 3.1 Asthma

Asthma is the most prevalent chronic condition in the paediatric population, associated with substantial adverse health consequences (11). Several factors have been associated with asthma onset, including low birth weight, preterm birth, respiratory viral infections, in-utero exposure to tobacco smoke, urbanisation, and various environmental or occupational exposures (12). Children and adolescents are particularly vulnerable to the harmful effects of environmental pollution due to their rapid physiological growth and development, making them more susceptible to respiratory disorders. In Slovenia, data on the prevalence of asthma among children and adolescents are limited; the available estimates date back to 2002 (13). Given the importance of environmental factors in asthma development and the lack of up-to-date prevalence data, a tool for collecting detailed environmental history data was developed prior to conducting a cross-sectional Health Interview Survey and Health Examination Survey (14). This study, the first national study capable of rigorously assessing the contribution of individual environmental risk factors to the onset and exacerbation of asthma in children and adolescents, is expected to be published in 2026. By integrating the observed health outcome, confirmed by a paediatrician, with environmental datasets, the study will generate high-quality evidence to support targeted interventions in both public health and clinical settings.

Future perspectives: atmospheric PM remains a major public health concern; however, regulating PM solely by mass concentration does not fully capture its health impacts. Including oxidative potential in air quality monitoring, as proposed in the revised European air quality regulations, provides a more accurate measure of PM’s harmful properties (15). Given the well-established role of oxidative stress in the pathogenesis of asthma, particularly in relation to PM2.5 exposure, greater efforts should be directed toward reducing oxidative stress as part of both asthma prevention and treatment strategies.

### 3.2 Sleep

Sleep disorders in children and adolescents are multifaceted, arising from a range of physiological, behavioural, psychological, and environmental factors. Understanding and addressing these disorders is essential for promoting healthy sleep habits and supporting optimal development in childhood and adolescence (16). However, there is a notable lack of national data on the prevalence of sleep-related problems among Slovenian youths. In addition, the tools currently used to assess sleep disorders, excessive daytime sleepiness, and chronotype or circadian preference in this population group have not been officially translated/validated in Slovenia, resulting in data of uncertain quality (17). Consequently, several established instruments were translated/validated. The results demonstrated strong content validity across the individual instruments, with particularly high validity indices. Translated versions also showed good content validity, confirming their suitability for both clinical practice and research on adolescent sleep quality in Slovenia (17).

Future perspectives: a recent review reported positive associations between various sleep problems and exposure to noise and light pollution, as well as heavy metals, second-hand smoke and air pollution (18). These findings underscore the need for a more nuanced approach to sleep health, integrating environmental factors into sleep disorder assessments. Given the importance of sleep quality for both mental and physical well-being, public health initiatives aimed at improving sleep hygiene and mitigating harmful environmental exposures should be prioritised as part of broader strategies to enhance the health and well-being of youths.

### 3.3 Systemic allergic reaction to bee venom

Exposure to repeated *Hymenoptera* stings (bees, wasps, bumblebees) is the leading environmental risk factor for developing an allergic reaction. Due to unavoidable seasonal or persistent exposure to the elicitors of *Hymenoptera* venom allergy, particularly bees, beekeepers represent a high-risk population group (19). However, national data on the estimated lifetime prevalence of first and recurrent systemic allergic reactions (SAR) to bee venom among Slovenian beekeepers, as well as the risk factors predisposing them to SAR, are lacking. Therefore, a nationwide cross-sectional study was conducted using a validated questionnaire (20), with clinician-confirmed observed health outcome. The study found a high lifetime prevalence of clinician-confirmed severe first and recurrent SAR to bee venom. Also, it identified several risk factors predisposing beekeepers to developing a first SAR to bee venom, including a history of a large local reaction, which was found to be novel. This was also the largest study of this kind in Europe, and possibly worldwide per capita, with the highest response rate reported to date (21).

Future perspectives: given that environmental factors and insect behaviour patterns can increase the risk of insect stings among beekeepers, and that many continue beekeeping even after experiencing their first SAR, further activities should focus on developing evidence-based recommendations to reduce and manage bee venom risks within this population group. This includes increasing the availability of epinephrine auto-injectors for emergency medical care, ensuring proper training for users, and providing regular refresher courses in allergic reaction management and auto-injector use.

## 4 CONCLUSIONS

This overview highlights key studies conducted at the Department of Public Health, Faculty of Medicine, University of Ljubljana, demonstrating meaningful progress in understanding how environmental exposures affect health across different population groups. The reviewed studies emphasise the importance of integrating environmental data into health research. Continued interdisciplinary collaboration, improved monitoring systems, and targeted prevention strategies will be essential to mitigate environmental health risks and support evidence-based policy development in Slovenia.
